# NetCooperate: a network-based tool for inferring host-microbe and microbe-microbe cooperation

**DOI:** 10.1186/s12859-015-0588-y

**Published:** 2015-05-17

**Authors:** Roie Levy, Rogan Carr, Anat Kreimer, Shiri Freilich, Elhanan Borenstein

**Affiliations:** Department of Genome Sciences, University of Washington, Seattle, WA 98195 USA; Department of Electrical Engineering & Computer Science, Center for Computational Biology, UC Berkeley, Berkeley, CA 94720 USA; Department of Bioengineering and Therapeutic Sciences, UCSF, San Francisco, CA 94158 USA; Newe Ya’ar Research Center, Agricultural Research Organization, Ramat Yishay, 30095 Israel; Department of Computer Science and Engineering, University of Washington, Seattle, WA 98195 USA; Santa Fe Institute, Santa Fe, NM 87501 USA

**Keywords:** Species interactions, Microbial ecology, Community assembly, Systems biology, Metabolic networks, Reverse ecology

## Abstract

**Background:**

Host-microbe and microbe-microbe interactions are often governed by the complex exchange of metabolites. Such interactions play a key role in determining the way pathogenic and commensal species impact their host and in the assembly of complex microbial communities. Recently, several studies have demonstrated how such interactions are reflected in the organization of the metabolic networks of the interacting species, and introduced various graph theory-based methods to predict host-microbe and microbe-microbe interactions directly from network topology. Using these methods, such studies have revealed evolutionary and ecological processes that shape species interactions and community assembly, highlighting the potential of this *reverse-ecology* research paradigm.

**Results:**

NetCooperate is a web-based tool and a software package for determining host-microbe and microbe-microbe cooperative potential. It specifically calculates two previously developed and validated metrics for species interaction: the *Biosynthetic Support Score* which quantifies the ability of a host species to supply the nutritional requirements of a parasitic or a commensal species, and the *Metabolic Complementarity Index* which quantifies the complementarity of a pair of microbial organisms’ niches. NetCooperate takes as input a pair of metabolic networks, and returns the pairwise metrics as well as a list of potential syntrophic metabolic compounds.

**Conclusions:**

The Biosynthetic Support Score and Metabolic Complementarity Index provide insight into host-microbe and microbe-microbe metabolic interactions. NetCooperate determines these interaction indices from metabolic network topology, and can be used for small- or large-scale analyses. NetCooperate is provided as both a web-based tool and an open-source Python module; both are freely available online at http://elbo.gs.washington.edu/software_netcooperate.html.

## Background

In the post-genomic era, genome scale reconstructions of various biological networks have become a powerful tool for studying the behavior of organisms [1]. For example, genome-scale metabolic models can be used to predict growth rates of microbial species following perturbation [2,3], signaling network models can be used to predict cell phenotypes [4], and regulatory networks can be used to map cell-specific developmental programs [5]. Methods for analyzing the topology of such genome-scale networks were shown to be especially useful for revealing systems-level properties and for identifying design principles of biological networks [6-8].

More recently, several such studies focused on using network-based analyses to predict ecological attributes of microorganisms, laying the foundation for a comprehensive *reverse-ecology* framework [9]. This framework focuses on the identification of topological properties in an organism’s metabolic network that may reflect adaptation to specific environments or ecological interactions. Borenstein *et al*., for example, exploited this principle to predict the effective chemical environments of numerous microbial species [10]. In this study, an algorithm was introduced to analyze the topology of a metabolic network to determine the set of exogenously acquired nutrients (termed the ‘*seed set’*) from which all other compounds in the network could be synthesized. Applying this algorithm to detect the seed sets of a large array of microbial species, several fundamental properties of the interface between organisms and their environments, as well as large-scale evolutionary trends, were identified. This reverse-ecology method was further highlighted for its biotechnological and environmental applications by numerous studies (e.g., [11-13]). The seed detection algorithm was later presented also as a web-based tool and a software package, making this method easily accessible to researchers with any level of computational expertise [14].

Importantly though, following the introduction of this reverse-ecology paradigm, several other techniques were developed, going beyond a single-species analysis and aiming to investigate multi-species microbial systems. Exploring such systems and characterizing the complex web of interactions between member species are crucial for gaining a principled understanding of these complex ecosystems, their assembly rules, and the fundamental principles that govern microbial ecology. These methods focused specifically on quantifying maximal cohabitation [15], effective competition [16,17], and niche overlap [18], providing a diverse set of tools for predicting species competitive potential.

Clearly, however, ecological interaction between species is not limited to competition and, in nature, cooperation between species represents an additional crucial aspect of species interaction, with potentially marked impact on both the interacting species and their environment [19]. Following the reverse-ecology paradigm and assuming that cooperative relationships are similarly reflected in the topology of metabolic networks, several metrics have recently been presented to predict the level of potential cooperation between interacting species. For example, Christian *et. al.* described a method to quantify the extent to which the set of metabolites a microbial species can synthetize in a *given* environment [20-22] is expanded by (or conversely, redundant with) the set of metabolic reactions carried out by an interacting partner [23]. This measure of *metabolic synergy* was used to demonstrate that metabolic networks that are neither too similar nor too dissimilar stand to gain the most from interaction [23] (and see also [19]). Here, we focus on two other topology-derived metrics that are specifically based on the reverse-ecology seed-finding algorithm described above to quantify the potential strength of cross-species ecological interactions by assessing the extent to which the biosynthetic capacity of one species can support or complement the nutritional requirements (*i.e.*, the seed set) of another species. The first metric, the *Biosynthetic Support Score* (BSS), quantifies the capacity of a host organism to meet the nutritional requirements of a parasitic endosymbiont [24]. This score has been used, for example, to predict the strength of interactions between eukaryotic hosts and potential pathogens, and revealed gradual adaptation of parasites to their specific hosts on an evolutionary scale. The second, the *Metabolic Complementarity Index* (MCI), quantifies the extent to which two microbial species may support one another through biosynthetic complementarity, and provides a measure of the *potential* for syntrophy that exists between them [18]. The Metabolic Complementarity Index has been used to determine community-level assembly rules in the human gut microbiome and to demonstrate that the microbiome is dominated by habitat filtering. Notably, these two metrics do not necessarily measure *active* parasitism or cross-feeding, but rather the metabolic *potential* for such interactions as reflected by the metabolic networks of the two organisms. The realization of this potential depends on the environment in which the two species are placed and the availability of nutrients in this environment ([25]; see also below).

To make these methods easily accessible to researchers of microbe-microbe and host-microbe interactions, here we present NetCooperate, a web-based tool and a software package for calculating both the BSS and MCI metrics. This tool provides a network-based framework for predicting cooperative species interactions, and complements previously introduced reverse-ecology tools in offering a comprehensive suite of network-based methods for predicting the ecological attributes and ecological interactions of microbial species.

## Implementation

**Figure 1:**
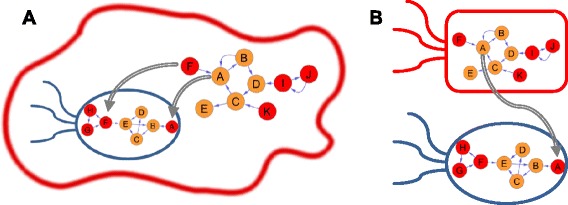
A schematic illustration of the Biosynthetic Support score and Metabolic Complementarity Index. **(A)** In this toy example, the blue bacterium represents a parasite that is supported by the red eukaryotic host. The metabolic networks of both species are shown. Seeds are colored red, whereas potential products are colored orange. In this example, the bacterium has 2 seed groups: A and F/G/H, both of which can be supported by the host metabolic network (grey arrows). Accordingly, the BSS of the host on the parasite is 1.0. **(B)** In this toy example of two interacting microbes, the complementation of the blue species by the red species is illustrated (for illustration purposes, the same metabolic networks as in panel **A** are used). The seed A of the blue species is a product of the red species and can therefore be complemented (grey arrow). However, since F is a seed in both species, the red species cannot complement F for the blue species. The MCI of the red species on the blue species is therefore 0.5.

NetCooperate receives as input the metabolic networks of two species, each encoded as a directed graph with nodes representing compounds and edges connecting substrates to products. It then calculates and plots the pairwise Biosynthetic Support Score and Metabolic Complementarity Index of each network to its partner. These metrics are based on pairwise comparison of species’ nutritional profiles as predicted by the seed detection algorithm [10]. Briefly, the seed detection algorithm utilizes a graph-theory-based method to analyze the topology of a metabolic network and to determine its seed set – the minimal set of compounds that should be acquired exogenously in order to allow the synthesis of all other compounds in the network. This set was shown to successfully serve as a proxy for the effective biochemical environment and the natural habitat of a species [10]. Importantly, due to the existence of multiple seed set solutions, this algorithm combines seed compounds into seed groups, such that any compound in the group can serve as an alternative seed in the seed set solution. Using the predicted seed sets of two species, the BSS is calculated based on the fraction of seed groups in the network of the first species (the *parasite*) of which at least one compound can be found in the network of the second species (the *host*; Figure 1A). Similarly, the MCI is calculated based on the fraction of seed groups in the network of the first species of which at least one compound can be found in the network, but *not* in the seed set, of the second species (Figure 1B). Notably, in calculating the BSS and MCI metrics, NetCooperate takes into account all possible seed set solutions. Moreover, the software further keeps track of the set of metabolites that were supported (or complemented) in each species. Both BSS and MCI range from 0 to 1, with 0 denoting no potential for cooperation and 1 denoting full cooperation. Importantly, in interoperating these scores there is no clear threshold for determining ‘cooperation’ vs. ‘no cooperation’, and instead, as demonstrated below, these metrics should be used in a comparative manner to assign physiological significance and to determine which species pairs exhibit strong potential for cooperation compared to other pairs in the same settings. Future studies could similarly use a comparative approach that is appropriate for the specific system under study.

**Figure 2:**
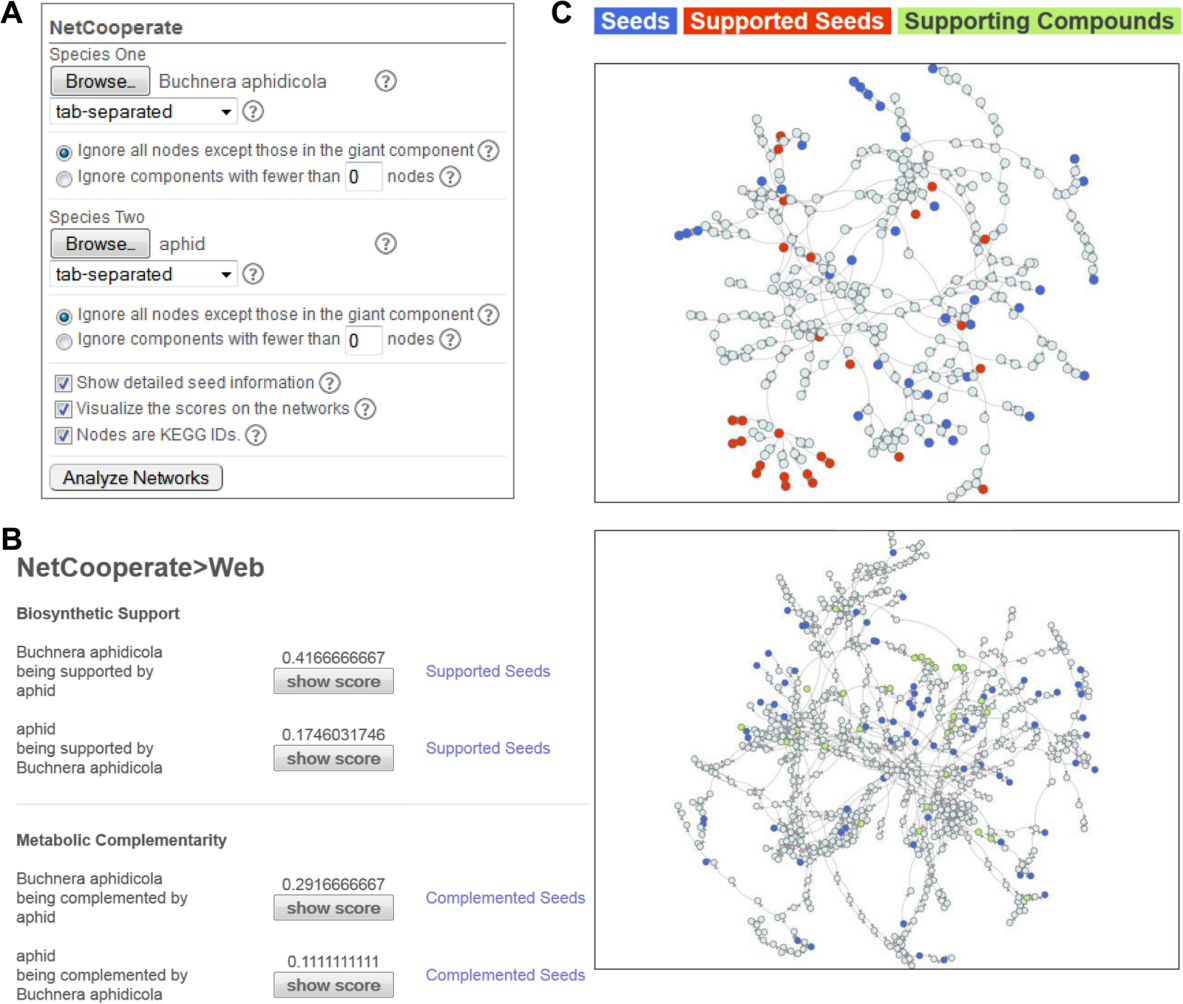
The NetCooperate web tool interface. **(A)** The data input panel. The user uploads two network files and selects analysis and visualization parameters. **(B)** The results panel. The BSS and MCI values are shown for all potential interactions. If the user selects ‘*Show detailed seed information’* the results include links to lists of the supported (or complemented) metabolites. **(C)** Network visualization. If the user selects ‘*Visualize the scores on the networks*’ both metabolic networks are plotted with seeds colored blue. Clicking on any ‘*show score*’ button will visually demonstrate compounds contributing the score: seeds which are supported (complemented) are colored red in the parasite network, and colored green in the host network.

The NetCooperate package was implemented in Python and allows users to quickly and easily integrate BSS and MCI calculation into existing software pipelines. The NetCooperate web-based tool is a CGI built on top of the NetCooperate Python module (Figure 2). The user supplies two networks, determines various threshold parameters for both, and can select several output options (Figure 2A). Input files can either be tab-, comma-, or space-delimited text files with each row representing a single directed edge, or tab-delimited text files representing network adjacency matrices. An explanation of each parameter may be found by hovering over the adjacent tooltip button (Figure 2A), as well as in the instruction manual available from the NetCooperate download page. The tool then calculates and displays the pairwise BSS and MCI metrics in both directions (Figure 2B). If the user selects *Show detailed seed information*, a list of the seed compounds that are present in the supporting network is linked to from the results. An option to display an interactive network visualization screen is additionally provided, allowing the user to view both networks, the status of each node, and metabolic information (Figure 2C). Finally, if nodes in the network are identified by KEGG compound IDs [26], the user may select the *Nodes are KEGG IDs* option, in which case the nodes in the network visualization serve as hyperlinks to KEGG database entries with detailed chemical descriptions of each compound.

## Results and Discussion

We have previously successfully utilized the cooperation metrics calculated by NetCooperate for studying a number of microbial systems and have shown that they provide tools for addressing fundamental questions in microbial ecology and evolution [18,24,27]. Such studies demonstrate the benefits of using systems-level tools and the impact such tools can have on elucidating global principles that govern multi-species systems. Specifically, below we discuss two such studies we have conducted that highlight the potential of NetCooperate and its applicability to several systems of interest [18,24]. These studies have promoted much interest and the application of this approach to address various challenges in biotechnological and medical settings has been highlighted [11-13]. It is our hope that providing the NetCooperate tool will enable the research community at large to apply this framework to a wide array of microbial ecosystems.

### Predicting host-parasite interaction and characterizing patterns of parasite adaptation

Parasitic species are clearly well adapted to their hosts. In introducing the Biosynthetic Support Score, Borenstein and Feldman aimed to examine whether such adaptation is reflected in the species’ metabolic networks and whether it can be used to predict parasitic species and specific host-parasite interactions [24]. To this end, they used the Biosynthetic Support Score to quantify the interaction between approximately 600 bacterial species and each of three model eukaryotic hosts (*human*, *fruit fly*, and *Arabidopsis*, representing a mammalian, insect, and plant host respectively). The distribution of BSS values of all bacteria against all hosts ranged from approximately 0.45 to 0.95. Importantly, a comparison of pathogenic bacteria to free living bacteria showed that parasitic bacteria have significantly higher BSS with all three hosts compared to free-living bacteria and that BSS was better in predicting parasitic species than classical metrics (e.g., genome size). Moreover, the BSS of a given parasite was higher when the model host was phylogenetically related to the parasite’s natural host (e.g., mammalian parasites had significantly greater BSS in human than in fruit fly), suggesting that the parasite’s metabolic network was sufficient to infer not only its parasitic life-style but also its preferred host.

To demonstrate the applicability of the Biosynthetic Support Score to evolutionary analysis, this study further integrated this cooperation score with phylogenetic analysis, calculating the BSS of both extant and ancestral species (obtained through phylogenetic reconstruction) within the phylum *Firmicutes*. It was then shown that the biosynthetic support provided by human to any given bacterium increased with the phylogenetic distance of the species from the common ancestor of *Firmicutes,* clearly demonstrating the gradual adaptation of parasites to their host environment on a global scale. Given the success of the BSS metric in predicting host-parasite interactions, it was later also proposed as a tool for designing culture media and for studying host-microbiome interactions [12,28].

### Assessing interaction between co-occurring microbes and elucidating assembly rules in the human microbiome

The human microbiome is a diverse and complex microbial ecosystem, with different individuals harboring markedly different sets of species. Previous surveys of the microbiome have revealed clear non-neutral patterns in the distribution of species and have demonstrated that certain species pairs tend to co-occur across microbiome samples whereas others tend to exclude one another [29]. Yet, the underlying forces that give rise to these patterns were not clear. The Metabolic Complementarity Index was first developed to address this challenge and to study emergent organizational properties of community assembly in the human microbiome [18]. This metric was first validated by predicting metabolic complementarity among several species of the human oral microbiota with well-characterized and assayed interactions [30], to confirm that it correctly identified preferred interacting partners. Indeed, in a series of controlled *in vitro* experiments, where microbes were placed in a nutrient-limited saliva medium, microbes were found to grow best in the presence of species with greater metabolic complementarity. In such settings, the ability of species to complement the nutritional requirements of their partners translates into active cooperation and improved growth. Moving on to *in vivo* communities of the human intestine, the MCI between all possible pairs among >150 gut dwelling microbial species was then calculated. By comparing the MCI among species’ co-occurring *partners* to their *excluders*, it was found that in fact in this nutrient-rich environment species with low MCI tended to co-occur, whereas species pairs with greater MCI excluded one another from a given host-habitat. This finding suggested that in the assembly of these communities habitat filtering outweighed the impact of species interaction and that species relied on the availability of nutrients in the environment rather than realizing the potential for cross-feeding [18,27]. Put differently, in this nutrient-rich environment, the potential for cooperation did not necessarily materialize and species assortment was based on the availability of nutrients in the environment rather than on the presence or absence of other species. An in depth analysis further revealed that not only is MCI not an artifact of phylogenetic relatedness, but that it was more successful at predicting species interactions. A similar analysis was used to investigate community assembly across multiple phylogenetic and biogeographic scales, demonstrating that metabolic complementarity had a greater influence on species co-occurrence patterns between members of the same phylum than across all species. Finally, applying the MCI to species co-occurrence across and within multiple body sites revealed that habitat filtering is a general assembly rule applicable to communities inhabiting heterogeneous anatomical sites within the human body.

## Conclusions

Network analysis has become an essential component in the study of microbiology. Metabolic, regulatory, and protein-interaction networks provide insight into the behavior and dynamics of individual cells [31-34], whereas ecological networks reveal processes defining the behavior of entire microbial communities [29,35,36]. Yet, molecular network properties are rarely used to explain patterns observed in ecological networks, although clearly, these two scales of organization are tightly linked. The reverse-ecology framework provides a powerful platform to address this challenge and to couple genomic information with environmental context. Specifically, the Biosynthetic Support Score and the Metabolic Complementarity Index represent two successful examples in which molecular network analysis can be applied to ecological studies of microbe-microbe and host-microbe interactions. Unfortunately, the implementation of such graph theory-based methods is not trivial, and may be beyond of the technical capabilities of microbiology researchers with no advanced computational skills. Above, we have presented NetCooperate, a web-based tool and Python package for easily performing the necessary computation. NetCooperate can be applied on a small-scale by those studying a microbe of interest, or it can be integrated into a larger workflow for large-scale analysis of entire communities. NetCooperate, along with previously introduced methods [14,17], completes the suite of reverse-ecology analysis tools accessible to researchers with any level of technical expertise.

## Availability and requirements

**Project name:** NetCooperate

**Project home page:**http://elbo.gs.washington.edu/software_netcooperate.html

**Operating system(s):** Platform independent

**Programming language:** Python

**Other requirements:** Python 2.7

**License:** GPL

**Any restrictions to use by non-academics:** For commercial use please contact the corresponding author

## Abbreviations

BSS: Biosynthetic Support Score
MCI: Metabolic Complementarity Index
